# CXCL8 Associated Dendritic Cell Activation Marker Expression and Recruitment as Indicators of Favorable Outcomes in Colorectal Cancer

**DOI:** 10.3389/fimmu.2021.667177

**Published:** 2021-05-07

**Authors:** Enhao Li, Xiaobao Yang, Yuzhang Du, Guanzheng Wang, David W. Chan, Di Wu, Peiqing Xu, Peihua Ni, Dakang Xu, Yiqun Hu

**Affiliations:** ^1^ Faculty of Medical Laboratory Science, Ruijin Hospital, School of Medicine, Shanghai Jiao Tong University, Shanghai, China; ^2^ Department of Obstetrics & Gynaecology, LKS Faculty of Medicine, The University of Hong Kong, Hong Kong, Hong Kong

**Keywords:** colorectal cancer, CXCL8-CXCR1/2, dendritic cell (DC), immune profiling, therapeutics

## Abstract

Accumulating evidence suggests that tumor-infiltrating immune cells (TICs) in the tumor microenvironment (TME) serve as promising therapeutic targets. CXCL8 (IL-8) may also be a potential therapeutic target in cancer. CXCL8 is a potent chemotactic factor for neutrophils, myeloid-derived suppressor cells (MDSCs) and monocytes, which are considered immunosuppressive components in cancer-bearing hosts. Here, we identified the TME-related gene CXCL8 in a high-ImmuneScore population that contributed to better survival in colorectal cancer (CRC) patients from The Cancer Genome Atlas (TCGA) database. An integrated gene profile and functional analysis of TIC proportions revealed that the dendritic cell (DC) activation markers CD80, CD83, and CD86 were positively correlated with CXCL8 expression, suggesting that CXCL8 may be functional as antitumor immune response status in the TME. The gene signature was further validated in independent GSE14333 and GSE38832 cohorts from the Gene Expression Omnibus (GEO). To test the differential contributions of immune and tumor components to progression, three CRC cell lines, CT26, MC38 and HCT116, were used. *In vitro* results suggested no significant growth or survival changes following treatment with an inhibitor of the CXCL8 receptor (CXCR1/2) such as reparixin or danirixin. *In vivo* treatment with danirixin (antagonists of CXCR2) promoted tumor progression in animal models established with CT26 cells. CXCR2 antagonism may function *via* an immune component, with CXCR2 antagonist treatment in mice resulting in reduced activated DCs and correlating with decreased Interferon gamma (IFN-γ) or Granzyme B expressed CD8^+^ T cells. Furthermore, CXCL8 induced DC migration in transwell migration assays. Taken together, our data suggested that targeting the CXCL8-CXCR2 axis might impede DC activation or recruitment, and this axis could be considered a favorable factor rather than a target for critical antitumor effects on CRC.

## Introduction

Colorectal cancer (CRC) is the second most commonly diagnosed malignant tumor worldwide (1). CRC is usually characterized by a lengthy and very complicated process, including multiple steps leading to the normal epithelium’s malignant transformation into cancer cells. It usually involves numerous genetic changes that lead to multiple phenotypic changes (2). In addition, chronic inflammation contributes significantly to tumor formation and progression. Inflammation seems to be a key process that mediates the relationship and dual functions of cancer cells and immune cells in the tumor microenvironment (TME). Current studies have evaluated the expression of various cytokines, chemokines and their receptors in CRC (3). Many studies have aimed to delineate the differential roles of cytokines, chemokines, and the TME in CRC relative to these components’ antitumor status through evaluation of subsets of immune cells.

Chemokines are signaling proteins secreted by cells that are crucial for mediating the recruitment of immune cells to inflammation sites. Chemotaxis involves the coordination of cells expressing appropriate chemokine receptors that can migrate along with chemokine concentration gradient, thereby promoting the localization of individual cells to specific tissues, such as tumor sites (4). Chemokines have dual functions: in T-cell-mediated reaction with tumor cells that play as antitumor immune responses, and in recruiting neutrophils, myeloid-derived suppressor cells (MDSCs) and monocytes to play immunosuppressive roles to promote tumor progression (4). C-X-C motif chemokine 8 (CXCL8), also known as interleukin 8 (IL-8), is mainly produced by macrophages. Furthermore, it is primarily responsible for neutrophil chemotaxis during the inflammatory process (5). CXCL8 is a chemokine whose signal transduction is mediated by the extracellular binding of two G-protein coupled receptors (GPCR), acting through CXC chemokine receptor type 1 (CXCR1) and CXC chemokine receptor type 2 (CXCR2) (6). The interaction between CXCL8 and CXCR1/2 regulates the trafficking of cells for cancer progression in the TME. Tumor-associated macrophages (TAMs) also express CXCR1 and CXCR2 (6), which led the CXCL8-CXCR1/2 axis to show the critical involvement for recruiting TAM to the TME and to play a crucial role in tumor immune escape (7). The CXCL8-CXCR1/2 interaction also regulates the processes of angiogenesis, tumor growth, proliferation as well as the survival of malignant cells (5). Numerous studies have shown that CXCL8 is expressed on endothelial cells, tumor-related macrophages and cancer cells, including CRC cells (8). In the sight of the clinical significance and biological function of the CXCL8-CXCR1/2 signal transduction axis in cancer, it has been suggested that CXCL8 and its receptors are considered as attractive targets for cancer treatment (6).

Overexpression of CXCL8 promotes the proliferation, migration and invasion of CRC cells. It is also closely related to CRC angiogenesis, metastasis, poor prognosis and poor disease-free survival (9). On the other hand, high expression of CXCL8 may protect against CRC liver metastasis, producing a good prognosis (10). Objectively, the role of CXCL8 is still controversial. This study confirms that CXCL8 is related to prognosis and suggests that high CXCL8 expression is associated with a better prognosis than low expression (11). However, there is controversy regarding the relevance of CXCL8 in cancer biology, which led us to investigate further whether chemokines in the CXCL8-CXCR1/2 signaling axis have a favorable role in the antitumor immune function or promote protumor behaviors.

Dendritic cells (DCs) are specialized antigen-presenting cells responsible for activating T cells, thereby coordinating the antitumor response (12). DC activation begins with taking up the antigens and migrating from peripheral tissues to the lymph organs. During antigen presentation, DCs upregulate the costimulatory receptor molecules CD86, CD80 and CD83 on their plasma membrane. CD86 is expressed during the early stages of DC activation. Besides, CD80 and CD83 are upregulated in activated DCs. Those makers have been established as hallmarks of DC activation during immune responses (13). DC activation is a crucial factor in the effective activation of T cells (14), whose various inflammatory mediators trigger in response to immune responses. However, the differential kind of specific inflammatory mediators and cytokines and chemokines correlated with DC activation in CRC are still unknown.

The immune and stromal classification of CRC can be linked to the molecular subtypes relevant to precision immunotherapy. In the current study, using the ESTIMATE algorithm to quantify the immune and stromal cellular components in tumors, we characterized the TME-related genes in CRC by functional enrichment analysis from CRC tissue specimens in The Cancer Genome Atlas (TCGA) database. We further identified that the TME-related gene CXCL8 and a high ImmuneScore contributed to better survival in CRC patients from the TCGA database. An integrated gene profile and functional analysis of tumor-infiltrating immune cell (TIC) proportions revealed that DC activation markers (CD80, CD83, CD86) were positively correlated with CXCL8 expression. Interestingly, we also identified the TME-related gene CXCL8 as contributing to better survival in CRC patients. Then, we used a small-molecule pharmacologic CXCR1/2 antagonist to block the CXCL8 receptor in murine tumor models established with CT26 or MC38 CRC cell to determine the effect of tumor-specific CXCL8-CXCR1/2 signaling impact on tumor behavior. In addition, we also investigated the role of host immune response by the therapeutic effects mediated through CXCR1/2 antagonism.

## Materials and Methods

### Gene Expression Profile Data and Clinical Parameters

The transcriptome of RNA-seq data and clinical parameters of CRC cases (normal samples, 41 cases; tumor samples, 473 cases) were downloaded from The Cancer Genome Atlas Program (TCGA) repository of the National Cancer Institute (https://cancergenome.nih.gov/). The data parameters were as follows: primary site (colon), data category with transcriptome profiling with gene expression and quantification with FPKM and counts, experimental strategy with RNA-Seq analysis and workflow type with HTSeq-FPKM and HTSeq-Counts. Default settings were used for the other filters.

In order to test and verify the discovery in the TCGA cohort, we downloaded GSE14333 and GSE38832 from the Gene Expression Omnibus (GEO) (https://www.ncbi.nlm.nih.gov/geo/). Expression series matrix files of both datasets were based on GPL570. The former included 290 samples of primary colorectal cancer patients, while the latter had expression data of 122 human samples. To get a larger size of samples, two cohorts were combined for analyses.

### Identification of Differentially Expressed Genes (DEGs) Between Tumor and Normal Tissues

The “edgeR” (15) R package (version 3.30.3) of the Bioconductor project in R software (version 4.0.2) was applied to discover the differentially expressed genes (DEGs). The genes expression with |log2 fold change (FC)| ≥3 and false discovery rate (FDR) <0.05 were considered to be significantly differentially expressed between tumor and normal tissues.

### Kyoto Encyclopedia of Gene and Genomes (KEGG) Pathway Enrichment Analysis

The KEGG pathway enrichment analysis was completed by the Database for Annotation, Visualization and Integrated Discovery (DAVID) database (http://david.ncifcrf.gov, version 6.8). Pathways with *p* Values <0.05 were considered statistically significant.

### Survival Test of Enriched KEGG Pathways Test

“GSVA” R package (16) (version 1.36.2) was applied to calculate single-sample GSEA (ssGSEA) scores of each pathway in each sample. The ssGSEA scores were used for the survival test of each pathway.

### Prediction of Stromal and Immune Components in Tumor Tissues

The ESTIMATE algorithm in the “estimate” R package (version 1.0.13) of R language version 4.0.2 was used to estimate the proportions of immune and stromal cellular components in the TME for each sample. A higher score estimated by the ImmuneScore or StromalScore indicated a larger number of immune or matrix cellular components in the TME. The ESTIMATE score was calculated as the sum of combination the ImmuneScore and StromalScore, representing the combined proportions of these two components in the TME (17).

### Identification of Differentially Expressed Genes (DEGs) in CRC

473 tumor tissue samples were divided into high-score and low-score groups based on separate comparison of the ImmuneScore and StromalScore to 0. The “edgeR” R package of the Bioconductor project of R software was applied to identify DEGs. The genes expression with a |log2 fold change (FC)| ≥1 and false discovery rate (FDR) <0.05 were considered to be significantly differentially expressed.

### Fraction of Tumor-Infiltrating Immune Cells (TICs)

Levels of immune cells infiltrating tumors were evaluated by CIBERSORT (18) (http://cibersort.stanford.edu/). As samples with a *p* Value >0.05 were ignored when analyzing TICs, 208 statistically significant samples were used for analysis.

### Cell Lines and Cell Culture

The colon carcinoma cell lines: MC38 Cell Line derived from C57BL6 murine colon adenocarcinoma cells, and CT26 is a murine colorectal carcinoma cell line from a BALB/c mouse. HCT116 is a human colorectal carcinoma cell line. All cells were cultured in RPMI 1640 with 10% FBS and 1% Penicillin-Streptomycin and were used within 10 generations. Mycoplasma contamination testing was performed to confirm that no mycoplasma contamination was present. Cells were cultured at 37°C with 5% CO_2_ under humidified conditions.

### Mice and Tumor Models

C57BL/6 and BALB/c mice were purchased from Charles River (Beijing, China). A total of 1×10^6^ CT26 or MC38 cells in 200 μl of PBS were implanted subcutaneously into the right flank region of each 6- to 8-week-old Balb/c (for CT26) or C57BL/6 (for MC38) mouse on day 0, and the treatment started on the same day after injection of tumor cells. Reparixin (HY-15251) and danirixin (HY-19768) were purchased from MedChem Express (MCE, NJ, USA) and dissolved in DMSO for storage. Reparixin and danirixin were dissolved in DMSO for storage. Reparixin was injected intraperitoneally at 15 mg/kg dissolved in a mixture of PEG300, Tween 80 and saline. Danirixin dissolved in PEG400 and ddH_2_O was administered by oral gavage at 15 mg/kg. All groups were treated every two days until the completion of the study. Tumor volume was measured every two days with a caliper and calculated as width^2^×length/2.

### Cell Proliferation Assays

The proliferation of CT26, MC38 and HCT116 cells was determined with a Cell Counting Kit-8 (CCK8; Dojindo, Japan) assay. Cells were plated in 96-well plates overnight and then treated with DMSO, reparixin or danirixin for 12, 24, 36, 48, and 60 h (37°C, 5% CO2). Ten microliters of CCK-8 solution were added to each well in the 96 well plates and incubated at 37°C for 1 hour. Cell proliferation was quantified by measuring the absorbance at 450 nm.

### Isolation of Immune Cells From Spleen

The mouse spleen was stripped and dipped in PBS solution. Spleen was ground to white through a nylon mesh screen in sterile PBS buffer and then filtrated. The solution was centrifuged at 300g for 5 minutes and the supernatant was discarded. 3ml of red blood cell lysis buffer (C3702, Beyotime, Shanghai, China) was used to lyse erythrocytes. Cells were washed then re-suspended in RPMI 1640 with 10% FBS and 1% Penicillin-Streptomycin. Live cells were counted using trypan blue dye exclusion (Sigma-Aldrich, St. Louis, USA) and diluted to 2 × 10^6^ cells/50uL for further use.

### DC Migration Assay

For transwell migration assays, freshly isolated mouse spleen cells were seeded in the upper well of a 24-well transwell plate (pore diameter 5 μm, Corning, NY, USA), which was pre-coated by matrigel (Corning). Lower chambers of the transwell were filled with 600 μl in the absence or presence of 10, 25, 50, 100 ng/ml of CXCL8 (200-08M, PeproTech), or 100ng/ml CXCL7 (300-14, PeproTech) as a negative control. To see whether the CXCR2 inhibitor can inhibit DC migration *in vitro*, 50 ul isolated immune cells from spleen and 25ug/ml danirixin (or vehicle control) were added in the upper chamber, while 600 ul CT26 cell culture supernatant fluids were added in the lower chamber. After 6 hours, the migrated cells were harvested from the lower chamber and further analyzed by flow cytometry.

### Flow Cytometry

Tumor tissue was immediately collected on the day when mice were sacrificed. Tumors were minced and digested with collagenase IV (Sigma-Aldrich, 300 U/mL) and a hyaluronidase solution (Sigma-Aldrich, 200 U/mL) in RPMI 1640 medium (Servicebio, Wuhan, China) supplemented with penicillin and streptomycin at 37°C for 1 hour. The cell suspension was centrifuged at 1,200 rpm for 5 min and mechanically dissociated through a nylon mesh filter. The antibodies used included anti-CD45-FITC (109806), anti-CD11c-PECy7 (117318), anti-MHC-II (IA/IE)-BV510 (107635), anti-CD8-PerCP-Cy5.5 (100734), anti-CD3-PE (100220), anti-CD19-APC (152409), anti-IFN-γ-APC (505810) and anti-GzmB-Pacific Blue (515407) purchased from BioLegend (San Diego, CA, USA). The Zombie NIR Fixable Viability Kit (BioLegend) was used to discriminate live and dead cells. To identify apoptotic and necrotic tumor cells after incubated with danirixin or reparixin, a BioLegend FITC Annexin V apoptosis detection kit with propidium iodide (PI) were used according to the manufacturer’s protocol. Data were acquired with an LSR-II system (BD Biosciences, Vianen, The Netherlands) and then analyzed with FlowJo software (version V10, Becton Dickinson, Ashland, OR, USA). To ensure single-cell gating, doublets were excluded with FSC-A and FSC-H linearity.

### Quantitative Real-Time PCR

Total RNA was extracted by TRIzol reagent (Invitrogen, Carlsbad, CA, USA). The concentration was analyzed by Nanodrop spectrophotometer (Thermo Fisher Scientific, MA, USA). PrimeScript RT Master Mix (TaKaRa, Japan) was used for reverse transcription. Mir-X™ RNA first-strand synthesis kit (TaKaRa) was used for transcribing and then for RNA expression analysis. SYBR Premix Ex Taq (TaKaRa) was used for real-time PCR in a 7900 HT Real-Time PCR system (Applied Biosystems, USA). The expression level of β-actin was used for endogenous control. The expression level was quantified using the 2-ΔΔct method. Primer information is accessible in **Supplementary Table 1**.

### Statistical and Survival Analyses

Data were analyzed using R software (version 4.0.2) and GraphPad Prism 8 software. One-way ANOVA and Student t test were used to determine significant differences, and *p* Values <0.05 were considered significant. Survival analyses were performed to assess the prognostic value of the indexes used in the study. The Kaplan-Meier (K-M) analysis was used to plot survival curves *via* the log-rank test.

## Results

### Identify Key Genes and Pathways in CRC

**Figure 1:**
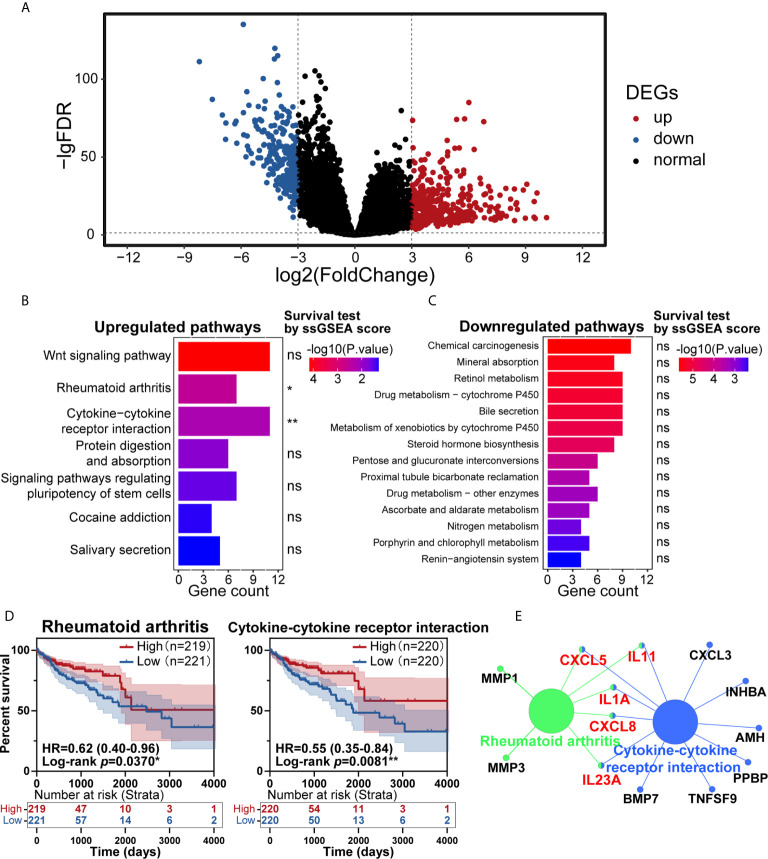
Identification of key pathways in colorectal cancer (CRC). **(A)** Analysis of differentially expressed genes (DEGs) of transcriptome profiling from The Cancer Genome Atlas (TCGA) cohort of CRC patients. **(B, C)** Kyoto Encyclopedia of Genes and Genomes (KEGG) pathway enrichment analysis of the DEGs in the TCGA cohort, with upregulated pathways **(B)** and downregulated pathways **(C)** shown. **(D)** Patients with CRC had significant survival differences between the high and low expression groups for rheumatoid arthritis (*p*=0.0370) or cytokine-cytokine receptor interaction (*p*=0.0081) (total patients n=440). **(E)** KEGG pathway enrichment analysis of the genes in the protein-protein interaction (PPI) module. ns: *p* > 0.05, *p < 0.05, **p < 0.01.

In the past few years, cancer’s immune classification has provided new ideas for the treatment of patients and prognostic and predictive factors for chemotherapy and immunotherapy (19). CRC has been a paradigmatic tumor for immune classification. We intended to identify the differentially expressed genes (DEGs) between tumor and normal tissues from patients, establish clinically relevant molecular subtypes, and identify significant CRC signatures (20). First, RNA-Seq data from the TCGA database were used to identify DEGs. Eventually, 620 DEGs were found, including 384 upregulated genes and 236 downregulated genes (**Figure 1A**). To explore these genes’ potential biological effects, Kyoto Encyclopedia of Genes and Genomes (KEGG) pathway analysis was then conducted using the DEGs from the dataset. KEGG analysis showed that the statistically significant 7 upregulated and 14 downregulated pathways were enriched in the CRC group (**Figures 1B, C** **)**. Then, the total 21 pathways with their ssGSEA scores were further filtered by survival analysis, and two upregulated pathways shown in **Figure 1D** (rheumatoid arthritis and cytokine-cytokine receptor interaction) were statistically significant by survival test. These two pathways were connected by CXCL8, CXCL5, IL11, IL1A and IL23A in PPI network analysis (**Figure 1E**), which was completed by ClueGo (version 2.5.7) in Cytoscape (version 3.7.1). The above results suggested that the highly expressed cytokine and chemokine components in tumors were relevant to prolonged survival in CRC patients.

### Identification of CXCL8 as a Key Regulator in Immune-Mediated CRC

**Figure 2:**
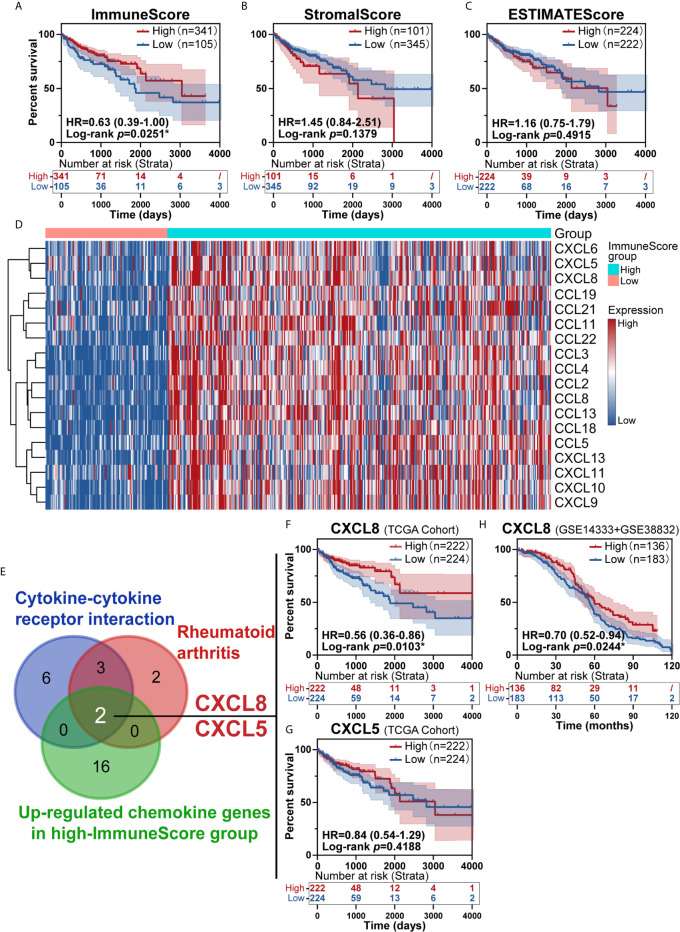
Identification of immune subtype genes. **(A)** Survival analysis of high and low scores for the ImmuneScore. **(B)** Survival analysis of high and low scores for the StromalScore (total patients n=446). **(C)** Survival analysis of high and low ESTIMATEScores. **(D)** Heatmap showed the levels of different chemokines in CRC patients classified into the high and low ImmuneScore groups. **(E)** Venn diagrams for rheumatoid arthritis or cytokine-cytokine receptor interaction pathway after filter survival analysis showing with overlap with upregulated chemokine genes from the high-ImmuneScore group. **(F, G)** The overlapping genes CXCL8 (**F**, *p*=0.0103) and CXCL5 (**G**, *p*=0.4188) identified with a K-M survival plot (total patients n=446). **(H)** The survival test for CXCL8 in GSE14333+GSE38832 confirmed the prognostic value of CXCL8 (total patients n=319, *p*=0.0244). *p < 0.05.

To investigate the prognostic value of the estimated proportions of immune and stromal cells in tumors, the ESTIMATE algorithm was employed to estimate the proportions of immune and stromal components in the TME for each sample. Higher scores estimated by the ImmuneScore or StromalScore represented larger amounts of immune or stromal cellular components in the TME. Here, we evaluated the associations of the ImmuneScore and StromalScore with overall survival. CRC patients were split into high- and low-score groups based on the ImmuneScore and StromalScore with a cut-off of 0. A high ImmuneScore value resulted in prolonged survival (**Figure 2A**), while the StromalScore was not related to the overall survival rate (**Figure 2B**), and a total ESTIMATEScore created by combining the ImmuneScore and StromalScore did not contribute to survival rates (**Figure 2C**). Furthermore, a high ImmuneScore was correlated with subset chemokines, including CXCL8 and CXCL5 through heatmap analysis (**Figure 2D**). We further assessed the genes from the rheumatoid arthritis and cytokine-cytokine receptor interaction pathways that were identified earlier (**Figures 1D, E**) to define overlapping subset chemokines that were enriched by ImmuneScore analysis (**Figure 2D**). Interestingly, the results indicated that CXCL8 and CXCL5 were genes in the overlapping enriched pathways (**Figure 2E**). To investigate the prognostic value of CXCL8 and CXCL5, we calculated the survival rates of patients in different subgroups. All tumor samples were divided into two groups based on the median expression of CXCL8 or CXCL5 and compared. High expression of CXCL8 was associated with a better prognosis (**Figure 2F**, *p*=0.0103). There was no survival advantage associated with CXCL5 expression (**Figure 2G**, *p*=0.4188). In order to further confirm the prognostic value of CXCL8 we discovered in TCGA cohort, we picked the corresponding probes of CXCL8 (202859_x_at) for survival test in GSE14333 and GSE38832 cohorts, which also showed a better prognosis in patients with higher CXCL8 expression (**Figure 2H**, *p*=0.0244). These results indicated that CXCL8 in the TME had a positive correlation with CRC patients’ overall survival rate.

### Dendritic Cells (DC) Activation Gene Signatures Were Positively Associated With CXCL8 Expression

**Figure 3:**
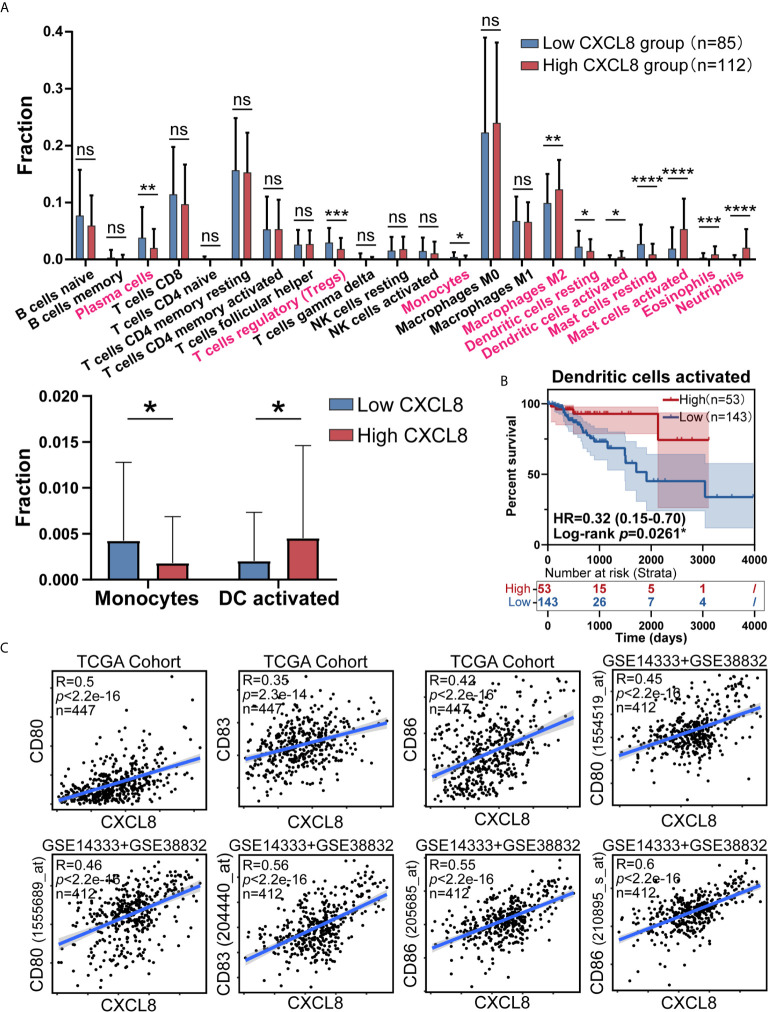
CXCL8 expression correlates with activated dendritic cells and their gene expression. **(A)** Comparison of immune cells fraction estimated by CIBERSORT between CXCL8 high and low group. **(B)** Survival test for more infiltrated activated dendritic cells (total patients n=196) with higher CXCL8 expression **(C)** Scatterplots representing the relationships between the expression of the dendritic cell activation genes CD80, CD83, and CD86 compared with CXCL8 expression in the TCGA cohort and GSE14333 and GSE38832 cohorts. ns: *p* > 0.05, *p < 0.05, **p < 0.01, ***p < 0.001, ****p < 0.0001.

As CIBERSORT returned the result, we analyzed the difference of cell fraction between CXCL8 high and low group, whose *p* Value of Student t test <0.05 was considered statistically significant. As shown in **Figure 3A** samples expressing higher CXCL8 had more infiltrated M2 macrophages, activated dendritic cells, activated mast cells, eosinophils, neutrophils and less infiltrated plasma cells, T cells regulatory (Tregs), monocytes, resting dendritic cells, resting mast cells. While MDSCs are known to be recruited by CXCL8, fraction of MDSCs is inaccessible in CIBERSORT algorithm, which limited us to study the potential effect of CXCL8 attracting MDSCs in TME through bioinformatic analyses (4, 18). Furthermore, significant immune cells were further filtered by survival test, in which only activated DCs had prognostic significance (**Figure 3B** and **Supplementary Figure 1**). According to previous studies, the TME in CRC contains a rich cytokine/chemokine milieu regulating tumorigenesis (21). Strikingly, the expression of DC activation gene signatures, such as CD80, CD83, and CD86 was positively correlated with CXCL8 expression (**Figure 3C**). Additionally, the correlation tests in GSE14333 and GSE38832 cohorts showed the similar result that CD80 (1555689_at and 1554519_at), CD83 (204440_at) and CD86 (205685_at and 210895_s_at) expressed higher as CXCL8 expression rose (**Figure 3C**). The estimates of immune cell population abundance across human tumors led us to hypothesize that CXCL8 might promote the recruitment of inflammatory cells, including neutrophils, mast cells, eosinophils, M2 macrophages, and especially activated DC, and thus CXCL8 improve patients’ prognosis through the recruitment of DC.

### CXCL8 Contributed to DC Recruitment in *Ex Vivo* Experiments

**Figure 4:**
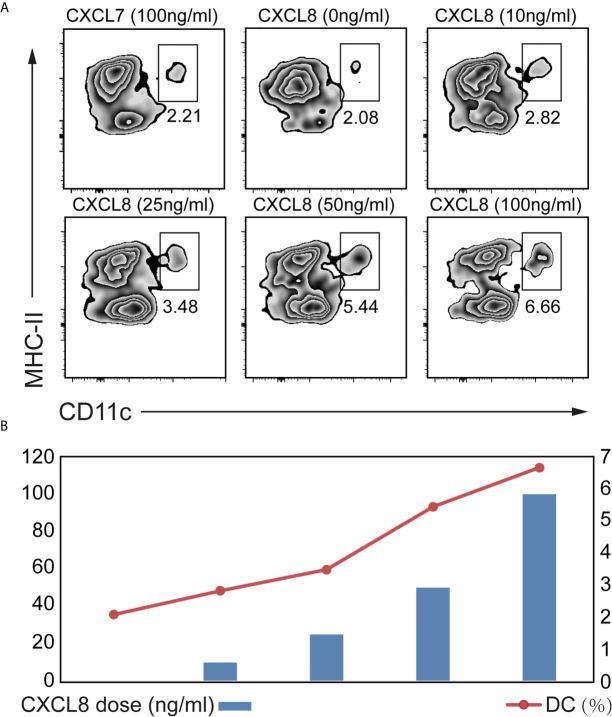
Determination of the CXCL8 chemokine that might attract DC. A transwell migration assay was performed. Splenocytes from mice were exposed to different concentrations of CXCL8 (0, 10, 25, 50 and 100 ng/mL) for 6 hours before quantifying DCs by flow cytometric analysis. **(A, B)** Flow cytometry was performed using CD11c and MHC-II gating strategy **(A)** to quantify DC **(B)**. Significant migration of DCs was observed upon treatment with CXCL8. For each treatment, the populations of DCs in the low- and high-dose groups were compared to the untreated group population.

To explore which cytokines/chemokines may contribute to the recruitment of DCs *ex vivo*, we compared the ability of CXCL8 to attract DCs among splenocytes isolated from mice. We also figured out that CXCL7 as an irrelevant chemokine didn’t contribute to DC attraction (**Figure 4A**). A significant (*p*<0.01) enrichment of DC was observed with both low (10 ng/mL) and high doses (100 ng/mL) of CXCL8 treatment using CD11c and MHC-II gating strategies for DCs (**Figure 4A**). Additionally, the results showed a significantly increased recruitment of DCs toward dose dependent manner (**Figure 4B**). Taken together, these results implied a robust association between DC infiltration and CXCL8 expression in CRC patients from (DC) activation gene signatures with CXCL8 expression.

### Antagonism of CXCR2 Promotes Tumor Progression *In Vivo*

**Figure 5:**
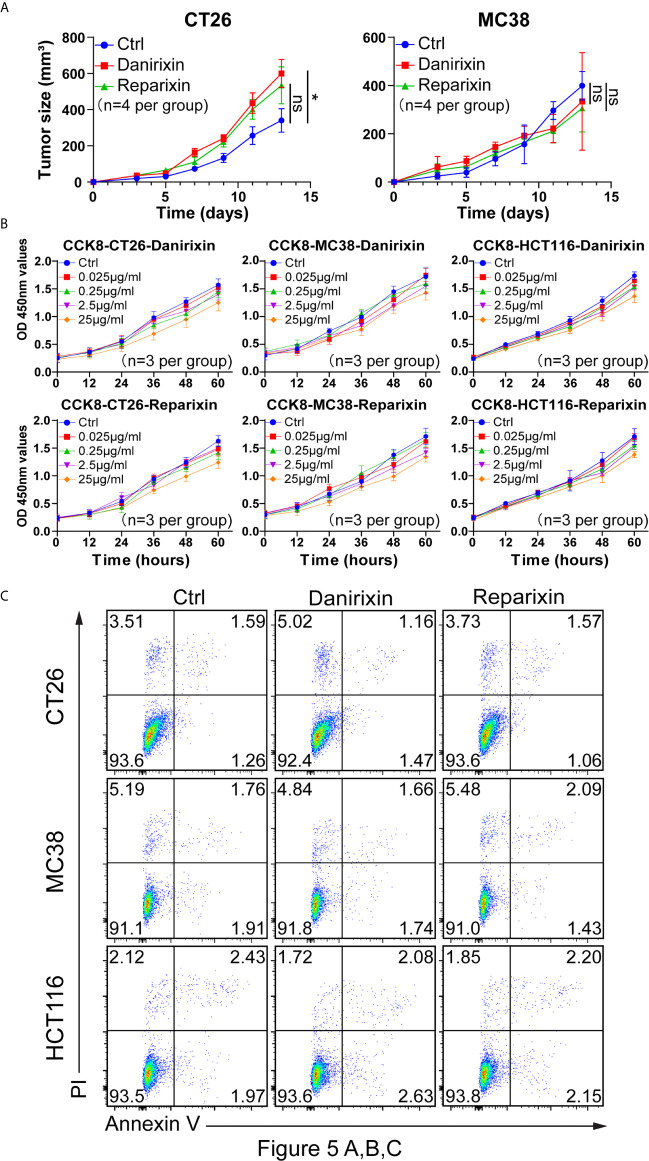
Antagonism of CXCR2 promotes the growth of CRC *in vivo*. **(A)** BALB/c mice were inoculated with CT26 cells, and C57BL/6 mice were inoculated with MC38 cells subcutaneously. The mice had been treated with DMSO (control), reparixin or danirixin for 2 weeks since the day tumor cells were inoculated. Mean and individual tumor volumes were measured every two days (n=4 per group). Tumor growth was evaluated by measuring tumor size and compared statistically by Student t test (*p < 0.05, ns: *p* > 0.05). **(B)** Proliferation of CT26, MC38 and HCT116 cells after reparixin or danirixin treatment as determined using a CCK-8 assay. Data are shown as the mean ± SEM. **(C)** Apoptosis of CT26, MC38 and HCT116 cells after reparixin or danirixin treatment as determined using FACS analysis of annexin V staining. All experiments were repeated at least three times.

From the above studies, we found that CXCL8 might affect antitumor activity through DCs. We further investigated whether CXCL8 could regulate tumor progression *in vivo*. Since CXCL8 is a ligand for CXCR1/CXCR2 and their expression levels are related, lack of CXCL8 (IL-8) in the mouse genome has been an essential limiting factor regarding the further investigation of targeting, so we examined the function of the chemokine receptor CXCR1/CXCR2 in two colon tumor models. To determine whether the CXCL8-CXCR1/CXCR2 axis would benefit antitumor activity, we employed a small-molecule antagonist of CXCR1/CXCR2, reparixin, and a small-molecule antagonist of CXCR2, danirixin. Danirixin were found to promote tumor growth in CT26 model, but not reparixin treated mouse in either CT26 or MC38 model (**Figure 5A**). Next, we questioned whether reparixin or danirixin treatment could affect tumor cell proliferation *in vitro*. It has been reported that inhibition of CXCL8 has been found to inhibit the migration of human colon tumor cell line (HCT116 cells) (22), so we selected HCT116 for our *in vitro* study. In CCK-8 experiments, the proliferation of reparixin- and danirixin-treated tumor cells did not change significantly (**Figure 5B**). In addition, reparixin or danirixin treatment did not obviously change the percentage of apoptotic cells (**Figure 5C**). Different immune behaviors found in CT26 and MC38 colon cancer model was mainly caused by higher immune infiltration in CT26 than MC38 (23). CT26 model also provided CXCR2 dependent mediated the trafficking and function of tumor-infiltrating immune cells in the TME (24). Taken together, these results indicated that targeting CXCR2 can promote tumor growth *in vivo* but had little or no direct effect on the proliferation of CRC tumor cells *in vitro*, which may be attributed to the difference between extracellular factors and intracellular factors in the tumor process.

### Antagonism of CXCR2 and Reduces DC and CD8^+^ T Cell Infiltration

**Figure 6:**
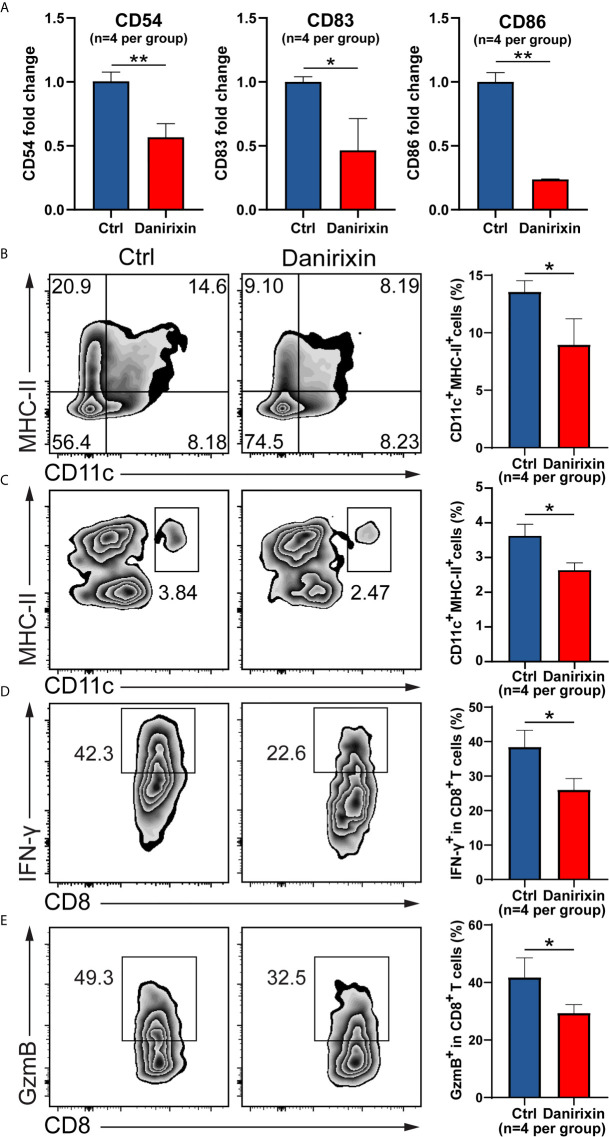
Antagonism of CXCR2 inhibits DC activation and prevents CD8^+^ T cell and DC infiltration in the TME. **(A–E)** CT26 murine colon tumor cells were subcutaneously injected into BALB/c mice. Since the day tumor cells were inoculated, the mice were treated with DMSO (control) and danirixin for 2 weeks. **(A)** The qRT-PCR analysis showed the statistical significance of three DC activation genes, CD54, CD83, CD86 between the group treated with danirixin and the control group. **(B)** Flow cytometric analysis of the tumor-infiltrated DC (CD11c^+^ MHC-II^+^) proportion in CT26 tumors after treatment with danirixin, **(C)** Flow cytometric analysis of DCs migration with or without danirixin treatment, **(D)** flow cytometry analysis of IFN-γ expressing CD8^+^ T cell and **(E)** Granzyme B-expressing CD8^+^ T cells in the TME of tumors generated with CT26 with or without treated with danirixin. Data are shown as the mean ± SEM. Statistical analyses were performed by a pairwise Student t test (n=4 per group). ns: *p* > 0.05, *p < 0.05, **p < 0.01.

Next, we were interested in which CXCL8 influences immune cell subsets in the regulation of antitumor immunity. It has been reported that *vivo* DC vaccine treatment on CT26 mouse, suggested that DC enhanced antitumor activities through the induction of CD8^+^ T cell and modulation of the tumor microenvironment (25). Using CT26 tumor tissues, we analyzed the DC activation genes by qRT-PCR, and the results supported the relationship between CXCL8 and DCs activation marker in TCGA cohort. As expected, expression of DCs activation genes such as CD54, CD83 and CD86 were declined significantly in the group treated with danirixin in comparison to the untreated group (**Figure 6A**). As expected, the percentage of CD11c^+^ and MHC-II^+^ DCs was significantly decreased in the antagonist groups compared to the control group (**Figure 6B**). To further validate our finding on danirixin inhibit DCs infiltration, transwell migration assays were carried out. Flow cytometric analyses showed a significant decrease of DCs among total splenocytes migrating toward CT26 cell culture supernatant fluids after treated with danirixin (**Figure 6C**). In addition, CXCR2 antagonist treatment reduced the IFN-γ expressing CD8^+^ T cells (**Figure 6D** **)**, as well as Granzyme B-expressing CD8^+^ T cells in the TME of tumors generated with CT26 mice (**Figure 6E**). Those subset CD8^+^ T cells expressing IFN-γ marker associated with Cytotoxic T lymphocytes (CTL), Granzyme B expression also serve as a signature of CTL activation, which can directly kill tumor cells and play a very important role in antitumor immunity. Additionally, in the complex tumor micro environment, CXCL8 as a chemokine may act as an antitumor factor through other subtypes of cells. Therefore, we analyse other subtypes of cells such as NK cells and MDSCs in CT26, which didn’t show any statistical difference between the treated group and the control group (**Supplementary Figure 4**). Thus, we can conclude that CXCL8-mediated antitumor effect mainly depends on activated dendritic cells instead of other types of immune cells. The results above brought us to the conclusion that antagonizing CXCL8-CXCR2 axis could inhibit the recruitment of DC in CRC tissue. All the results collectively indicated that antagonism of CXCR2 could impacted functional CD8^+^ T cell and DC infiltration into tumor sites in the CRC mouse model, which indicated that these treatments could change the TME in the CRC mouse model into a worse situation.

## Discussion

In the era of targeted therapy, especially immunotherapy that relies on the composition of the TME, we are committed to integrating the molecular and immune classification of CRC by solving the problems related to inflammation and the immune cell composition of molecular subtypes of CRC. To achieve this purpose, we applied a computational ESTIMATE algorithm to infer the abundances of immune cell populations and cytokines/chemokines from transcriptomic data. Using this method, we found that a high ImmuneScore value produced better survival. Among the chemokines upregulated in high-ImmuneScore group, CXCL8 had a prognostic value. Afterwards, We first identified that CXCL8 expression was correlated with the DC subtype compared with the other subtypes. CXCL8 was also associated with DC activation and recruitment, which indicated a favorable outcome for CRC. Treatment with a small-molecule antagonist of CXCR1/2, the receptor of CXCL8, was demonstrated to promote tumor progression in animal models established with CT26 cancer cells. Therefore, Targeting CXCL8 and the CXCR2 axis as a therapeutic strategy might impede DC activation or recruitment, which could lead to the opposite effect of antitumor immunity.

A few clinical data have indicated that the elevated levels of CXCL8 in patients’ serum or tissues from a cohort study of patients with CRC related to clinical characteristics such as grade, stage and metastasis. CXCL8 is secreted by monocytes and macrophages, which exerts potent angiogenic properties on endothelial cells through interaction with CXCL8 receptors CXCR1 and CXCR2 for regulating angiogenesis in colorectal cancer (26). Recent studies have documented that CXCL8 promoted migration, invasion, and proliferation of human cancer cells through EMT induction through the PI3K/AKT/NF-κB signaling axis (6, 27). Besides, CXCL8 is a potent chemotactic factor for neutrophils, MDSCs and monocytes, which are considered immunosuppressive components in cancer-bearing hosts (28, 29). All these data have identified CXCL8 as a poor prognostic marker that can be a promising therapeutic target.

However, little was known about the significance of CXCL8 expression correlated to the antitumor immunity induced by functional DCs. DCs are essential cells for generating antitumor immunity, but tumors can disable DCs through either to avoid immune recognition or to disable effector T-cells for escaping immune surveillance. DC activation can be induced and tested by conditioned media from number of colorectal cancer (CRC) cell lines, as reported by Michieisen AJ et al. (30). In a study, tumor conditioned medium (TCM) from a CRC line inhibited five DC markers: MHC-II, CD80, CD83, CD86 and CD54 (31). Increased expression of some activation markers on dendritic cell surface stimulates T cell activation with an antitumor immunity functional response (12). CD83 is one of the most characteristic cell surface markers of fully activated DC, which can enhance the T cell stimulation ability of DC. CD86 and CD80 are also costimulatory molecules binding to T cells while expressing a high level of MHC class II and can present antigens to the T cells (32). Altered MHC II ubiquitination reduces the ability of DC to present antigen in many types of cancer, a mechanism by which tumor cells evade T cell responses (33).

Our study revealed that CXCL8 was a good prognostic biomarker, since it contributed to DCs recruitment and related to DC activation. First, we found that a high ImmuneScore was correlated with prolonged survival, which indicated that favorable factors such as certain cytokines, chemokines and immune cell subsets played important roles in antitumor immunity. Second, CXCL8 was identified as a key regulator of immunity in CRC by many overlapping signaling pathways. Third, CXCL8 was found to have prognostic value compared with other cytokines and chemokines. Fourth, CXCL8 was associated with DC activation and recruitment. Fifth, targeting the CXCL8-CXCR1/CXCR2 axis by antagonizing CXCR2 promoted tumor progression *in vivo* by impeding DC activation or recruitment, leading to the opposite effect of antitumor immunity. Thus, CXCL8 might be considered a favorable factor for significant antitumor effects in CRC-bearing hosts, at least in our study.

Since *in vitro* DC activation experiments may not reflect a patient’s tumor DC activation status, we did not perform such experiments; instead, we assessed DC activation markers in larger cohort samples. We found that CXCL8 expression was positively correlated with the expression of DC activation genes such as CD80, CD83, and CD86 (**Figure 3C**). Furthermore, higher infiltration of activated DCs was strongly linked with an increased survival rate in CRC patients, to a similar extent as elevated CXCL8 expression. In addition, DC activity and migration were analyzed by FACS in CT26 tumor tissues. In targeting the CXCL8-CXCR1/CXC2 axis, the lack of CXCL8 in the mouse genome limited us to targeting CXCL8 to antagonize CXCR1/2 instead of CXCL8. Antagonism of CXCR2 could effectively prevent functional CD8^+^ T cells and DCs activation from infiltrating tumor sites in a CRC mouse model, which could change the CRC mouse’s antitumor immunity model to promote tumor progression rather than regression.

We found that the alter of the immune behavior was only found in CT26 colon cancer model, not in MC38 mice, mainly because CT26 had the higher immune infiltration than the MC38 model (23). Additionally, antagonism of CXCR2 (danirixin) could promote tumor progression more effectively than CXCR1/2 (reparixin) in CT26 tumor model. Furthermore, main reasons why reparixin and danirixin behavior different on the antitumor role are as follows. 1) CXCL8 (IL-8) and its receptor CXCR2 in the tumor microenvironment for prompted colon cancer progression and metastasis have been reported (8), which was opposite to our findings of suppression tumor progression. However, that study have suggested that the regulation of IL-8 within the tumor and microenvironment play a critical role, but the impact of tissue microenvironment-derived CXCL8 and CXCR2 to date remains difficult to evaluate. Since they targeted the cancer cell rather than immune cells, the mouse colon carcinoma cell line CT26 and human CRC cell line HCT116 confirmed the high expression of CXCR2 in the two cell lines. 2) Interestingly, Mice bearing MC38 tumor cells treated with CXCR2-transduced pmel-1 T cells showed enhanced tumor regression and survival compared with mice receiving control, which implicated that the introduction of the CXCR2 gene into tumor-specific T cells could enhance their localization to tumors and improve antitumor immune responses, and it was correlated our results with CXCR2 mediated DC to suppression role in tumor growth (34). In addition, it has been reported that increased tumor-infiltrating DCs and upregulated their CD86 expression *in vivo* lead to increased tumor-infiltrating CD8^+^ T cells and enhanced PD-L1 and MHC class I expression on tumor cells (35). More importantly, in CT26 colon cancer–bearing mice, the combinational use of imiquimod (TLR7) with autologous GVAX therapy displayed increased expression levels of DC marker CD86 and CD9, which led to decreased Foxp3^+^ regulatory T cells in TDLNs and increased CD8^+^ T cells which correlate with an antitumor phenotype (36). Collectively, these findings indicate that activate DCs as a positive regulator of T-cell priming, could enhance the immunologic antitumor effects.

As we discussed above CXCL8/CXCR2 had a role in DC-mediated anticancer immunity remains unclear, and all depend on targets specific for CXCL8, CXCR1/CXCR2, or CXCR2 alone. The different behaviors of reparixin or danirixin depend on the specific target, such as targeting CXCL8, CXCR1/CXCR2 or CXCR2, or depend on the specific target cells, such as tumor cells or different immune cell subpopulations. The efficacy of inhibition of CXCR2 has been widely investigated in preclinical experiments. Current inhibitors of CXCR is well discussed, and CXCR2 antagonists have been initially considered administrated in respiratory diseases and gradually in cancer for more insights into tumor microenvironment. Reparixin is a non-competitive CXCR1/2 antagonist. It has shown in a phase Ib trial for HER-2-negative metastatic breast cancer. Danirixin, another oral selective CXCR2 antagonist, has been investigated in patients with virus infection disease (influenza), which had small-sample clinical trial (NCT02469298). CXCR2-mediated neutrophils play an important role in anti-infection and control pathogen invasion (37).Current evidence suggests that TICs in the TME serve as promising therapeutic targets. It has also been proposed that CXCL8 may be considered as a therapeutic target for cancer treatment. CXCL8 is a potent chemotactic factor for neutrophils, MDSCs and monocytes, leading to immunosuppression. Our data suggested that targeting the CXCL8-CXCR1/2 axis as a therapeutic strategy might impede DC activation or recruitment. This axis could be considered a favorable factor for important antitumor effects in CRC rather than a target. Considering the multiple mechanisms used by tumors to evade the immune response, the use of modified DCs for cancer immunotherapy has achieved promising results. Experiments investigating DC immunotherapy using mouse models have shown that the growth rate of tumors is reduced, and the number of metastases is diminished. The survival rate of tumor-bearing animals is increased and the tumor-specific cytotoxic T lymphocyte (CTL) response is initiated (38, 39). Despite proven immunotherapies as a favorable safety profile, antitumor DC-based treatment has not succeeded with significant objective of clinical responses in clinical trials (40). A rational reason is that the low efficacy of DC vaccines may be related to the fact that the human immune system is suppressed to a relatively great extent by tumors. We need to think about the reasons for these impacts, such as 1) the chemotactic properties of DCs in response to specific chemokines being strictly regulated during DC development, 2) MHC I/II molecules on the DC surface and the weak polarization of antitumor immune responses, and 3) DC activation markers and activation status in tumors. Generally, CXCL8-CXCR1/2 axis-associated DC activation and recruitment might indicate the modulation of the DC system at the tumor site by the cytokine and chemokine network.

## Conclusions

In summary, it was previously reported that CXCL8 acts as a key multifunctional chemokine to modulate tumor cells for proliferation, invasion, migration, and as a chemotactic factor for neutrophils, MDSCs and monocytes, which served as immunosuppressive components in cancer-bearing hosts and led to a poor prognosis. CXCL8 is considered a potential attractive target for cancer treatment. However, our data suggest that CXCL8 is a key regulator of immunity in CRC that acts through associated DC activation gene expression, probably indicators of favorable outcomes of this particular subtype of CRC. Targeting the CXCL8-CXCR1/CXCR2 axis by antagonizing CXCR2 promotes tumor progression *in vivo* by impeding DC activation or recruitment, leading to the opposite effect of antitumor immunity. The CXCL8-CXCR2 axis could be considered a favorable factor for important antitumor effects on CRC rather than a target.

## Data Availability Statement

The original contributions presented in the study are included in the article/**Supplementary Material**. Further inquiries can be directed to the corresponding authors.

## Ethics Statement

The animal study was reviewed and approved by Faculty of Medical Laboratory Science, Ruijin Hospital, School of Medicine, Shanghai Jiao Tong University.

## Author Contributions

YH and DX contributed to the concept and designed the research work. EL, XY, YD and GW performed the experiments, acquisition, analysis and interpretation of the data. EL, XY, YD, YH and DX drafted the article. PX, PN, DW, DX and YH provided critical reagents and supervised the research. All authors contributed to the article and approved the submitted version.

## Funding

This work was supported by grants from the National Natural Science Foundation of China (NSFC) (81871274, 81871715, 82071811 and 31670905).

## Conflict of Interest

The authors declare that the research was conducted in the absence of any commercial or financial relationships that could be construed as a potential conflict of interest.
