# Supplementary material for: CXCL8 Associated Dendritic Cell Activation Marker Expression and Recruitment as Indicators of Favorable Outcomes in Colorectal Cancer
Source: Front Immunol. 2021 May 7;12:667177. doi: 10.3389/fimmu.2021.667177 (PMC8138166; doi:10.3389/fimmu.2021.667177)
Supplement: Supplementary file 1 [file DataSheet_1.pdf]

Supplementary Figure 1

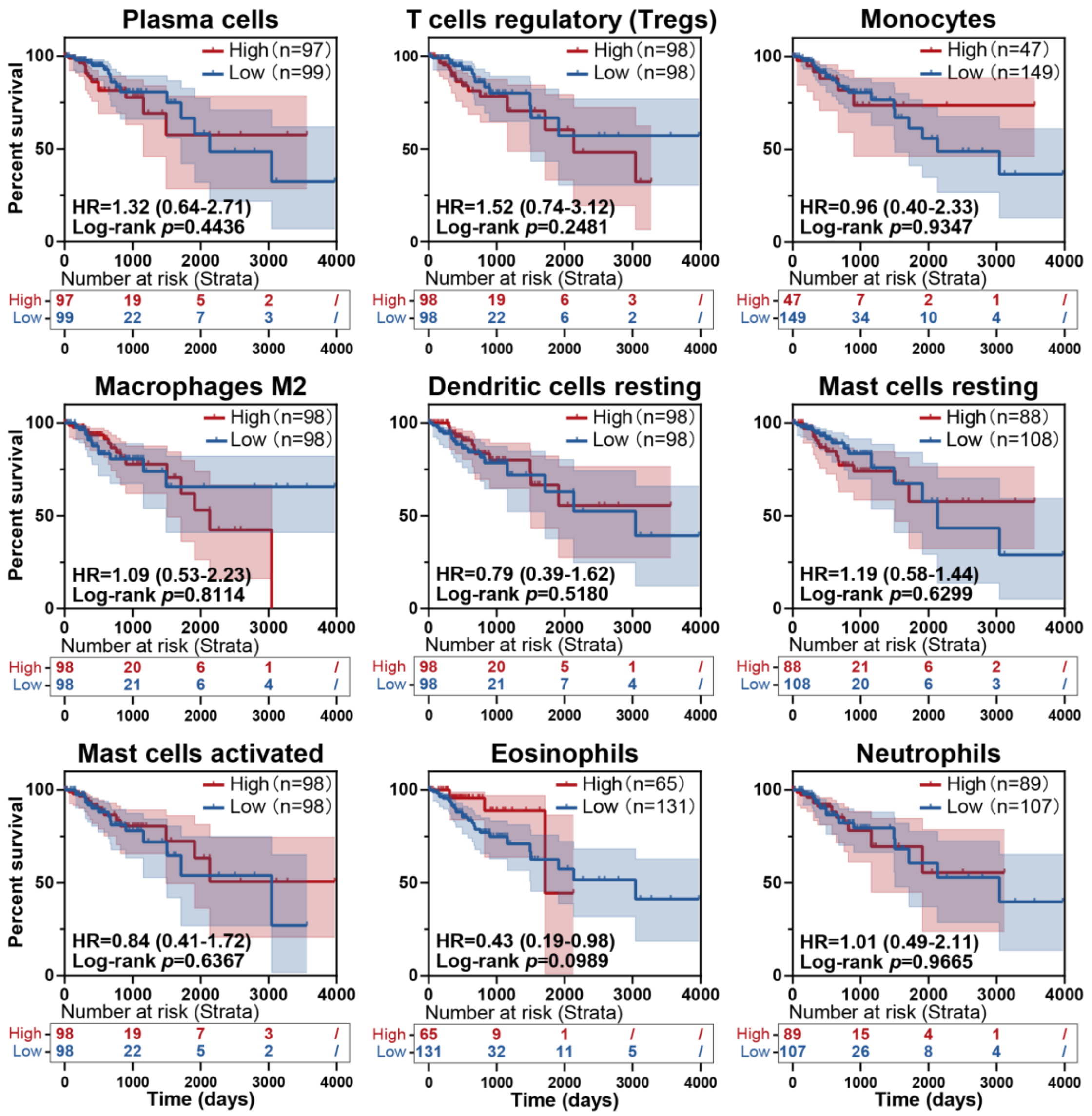

Figure S1

Figure S1. Survival curves of other significant types of cells aside from activated DCs. K-M plots of significant cells from more infiltrated M2 macrophages, activated mast cells, eosinophils, neutrophils and less infiltrated plasma cells, T cells regulatory (Tregs), monocytes, resting dendritic cells, resting mast cells in high CXCL8 group (total patients n=196).

Supplementary Figure 2

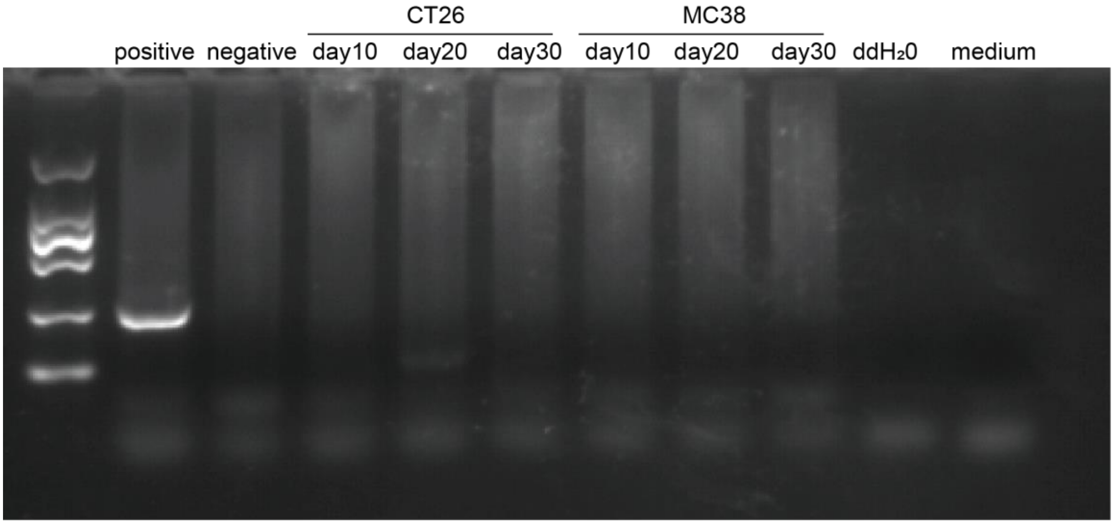

Figure S2

Figure S2. Mycoplasma contamination detection results. The culture medium supernatants of CT26 and MC38 at day 10, 20 and 30 were collected and centrifuged. Positive control (Mycoplasma) and negative control (ddH_2O and the medium) were set. Relevant primers were used for accounting amplification and gel electrophoresis. (Primer information is accessible in Supplementary Table 1). If the fragment of about 280bp can be amplified, the result is positive. The cells used are shown free of mycoplasma contamination.

Supplementary Figure 3

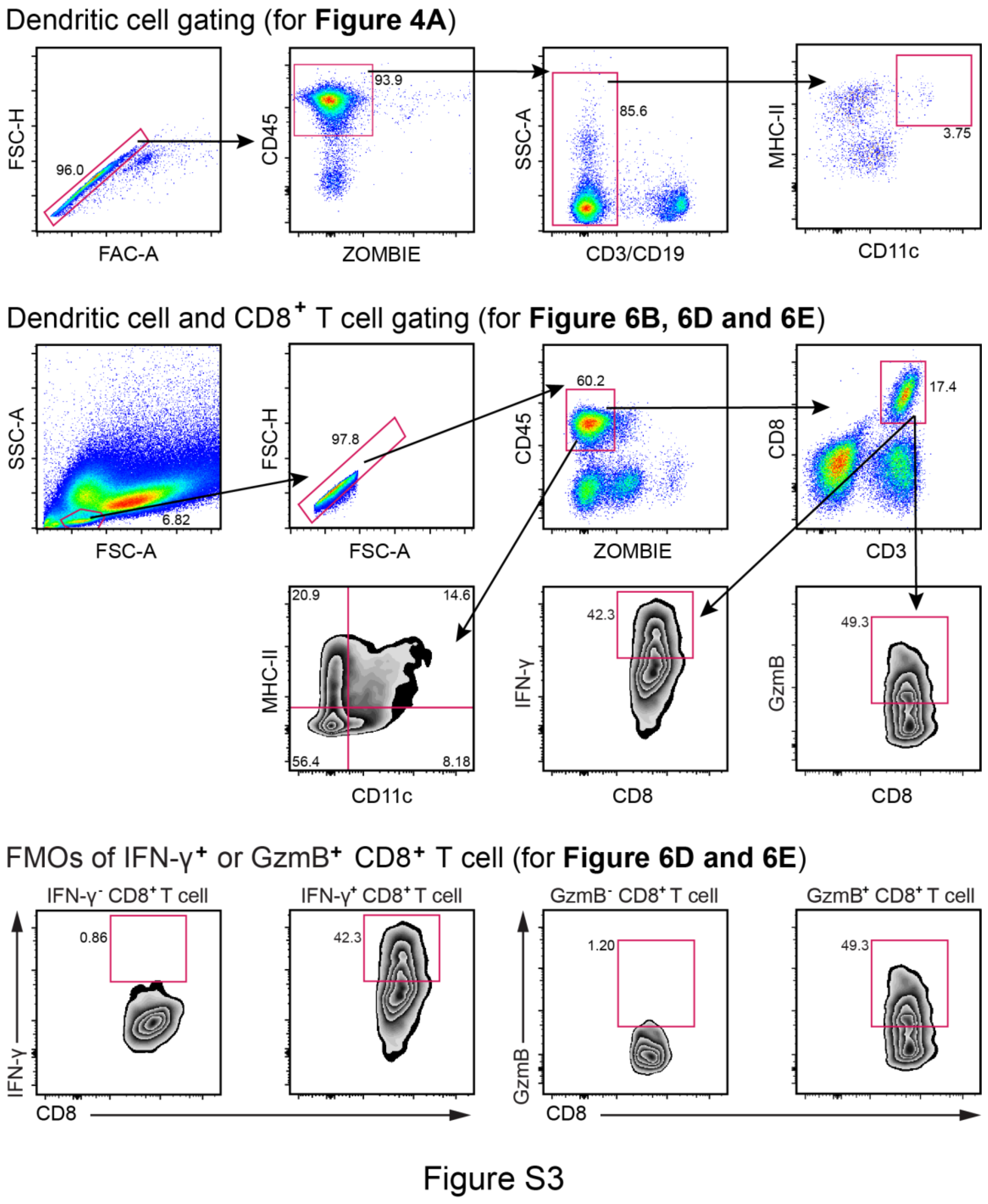

Figure S3. The gating strategy for DCs and CD8^{+} T cells (Figure 6B-C), and the FMOs used to determine these gates of IFN-γ^{+} or GzmB^{+} CD8^{+} T cells as well.

Supplementary Figure 4

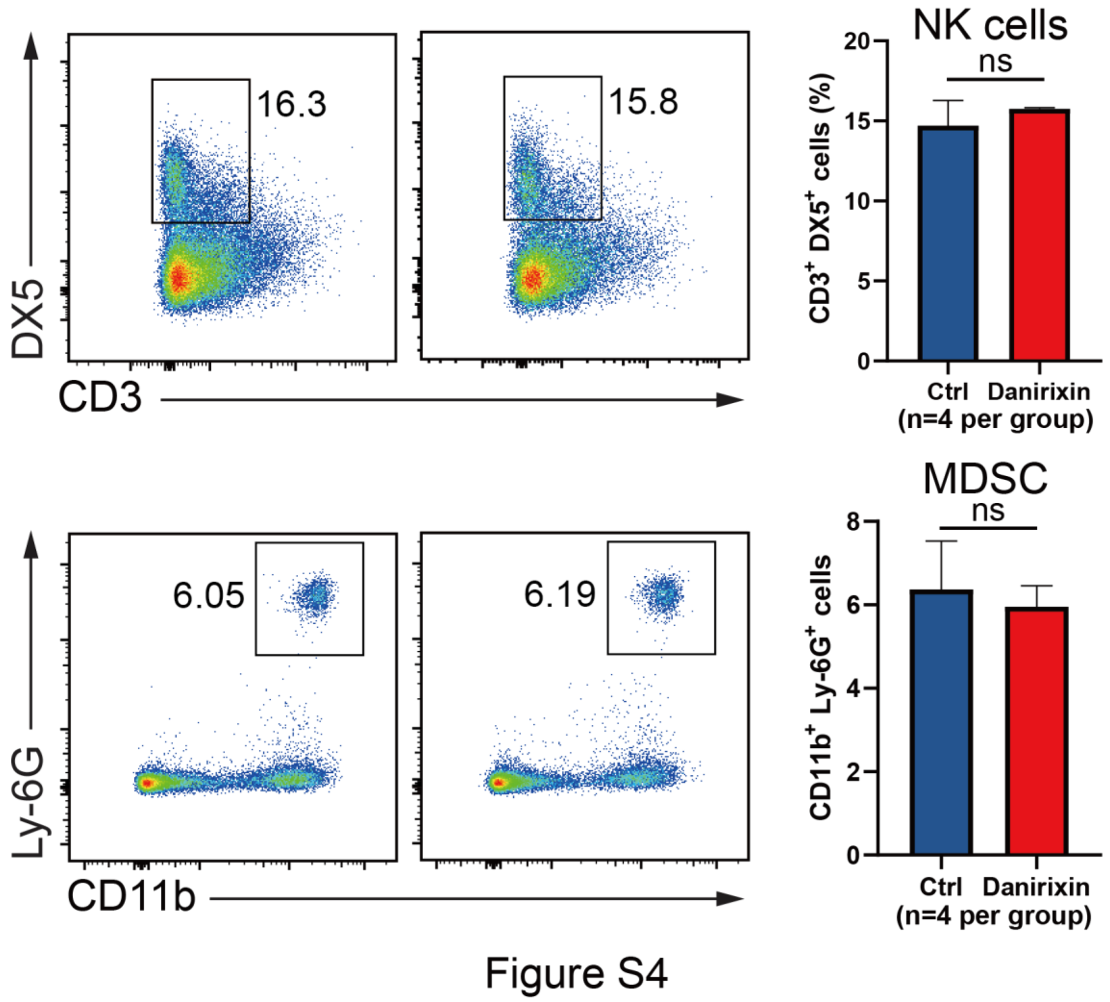

Figure S4. Identification of DCs as the dependence of CXCL8-mediated antitumor effect. In order to figure out whether CXCL8-mediated antitumor effect is also dependent on other types of immune cells in TME, we analyzed NK cells and MDSCs in CT26, which showed no statistical significance between the group treated with danirixin and the control group. Therefore, we have come to the conclusion that CXCL8-mediated antitumor effect mainly depends on activated DCs.
