# Supplementary material for: CXCL8 Associated Dendritic Cell Activation Marker Expression and Recruitment as Indicators of Favorable Outcomes in Colorectal Cancer
Source: Front Immunol. 2021 May 7;12:667177. doi: 10.3389/fimmu.2021.667177 (PMC8138166; doi:10.3389/fimmu.2021.667177)
Supplement: Supplementary file 2 [file Table_1.pdf]

Table S1 Primers used in the experiments.

Gene	Primer	Sequence (5′-3′)
**Primers for qRT-PCR**		
ICAM-1	forward	GCCTGGCATTTCAGAGTCTGCT
	reverse	AAACCAGACCCTGGAACTGCAC
CD83	forward	ATGAGCTCCATCCTCAGATGGCAA
	reverse	AAAGCACTCACGAGGTTGACCAGA
CD86	forward	TCTCCACGGAAACAGCATCT
	reverse	CTTACGGAAGCACCCATGAT
**Primers for Mycoplasma**		**Sequence (5′-3′)**
Mycoplasma		GGGAGCAAACAGGATTAGATACCCT
		TGCACCATCTGTCACTCTGTTAACCTC
